# Frequency of internet use, economic income, and health of the population—comparative analysis of urban and rural areas based on Chinese General Social Survey

**DOI:** 10.3389/fpubh.2024.1475493

**Published:** 2024-10-17

**Authors:** Xifeng Yang

**Affiliations:** International Education College of Jiangxi Science and Technology Normal University, Nanchang, China

**Keywords:** internet use, economic income, resident health level, urban–rural differences, healthy China

## Abstract

People’s health is one of the important supports for China’s economic development. This study uses the 2021 Chinese General Social Survey (CGSS) data to empirically test the impact of residents’ Internet use frequency and economic income on residents’ health level, and analyzes the differences between urban and rural areas. The empirical test results show that, firstly, the frequency of Internet use can promote the health level of residents, and the promotion effect of Internet use frequency on the health level of rural residents is higher than that of urban residents; Secondly, economic income has a positive promoting effect on the health level of the entire sample of residents, but in urban samples, the regression between economic income and residents’ health level is not significant. Third, in the heterogeneity test of region, gender and age, it is found that the impact of residents’ Internet use frequency and economic income on residents’ health level also has urban–rural differences. Based on this, this study suggests that the government can continue to make efforts to further promote the health level of residents by improving the Internet penetration rate, strengthening the use of Internet skills, carrying out Internet professional skills training and promoting the high-quality development of Internet content.

## 1 Introduction

**Figure 1:**
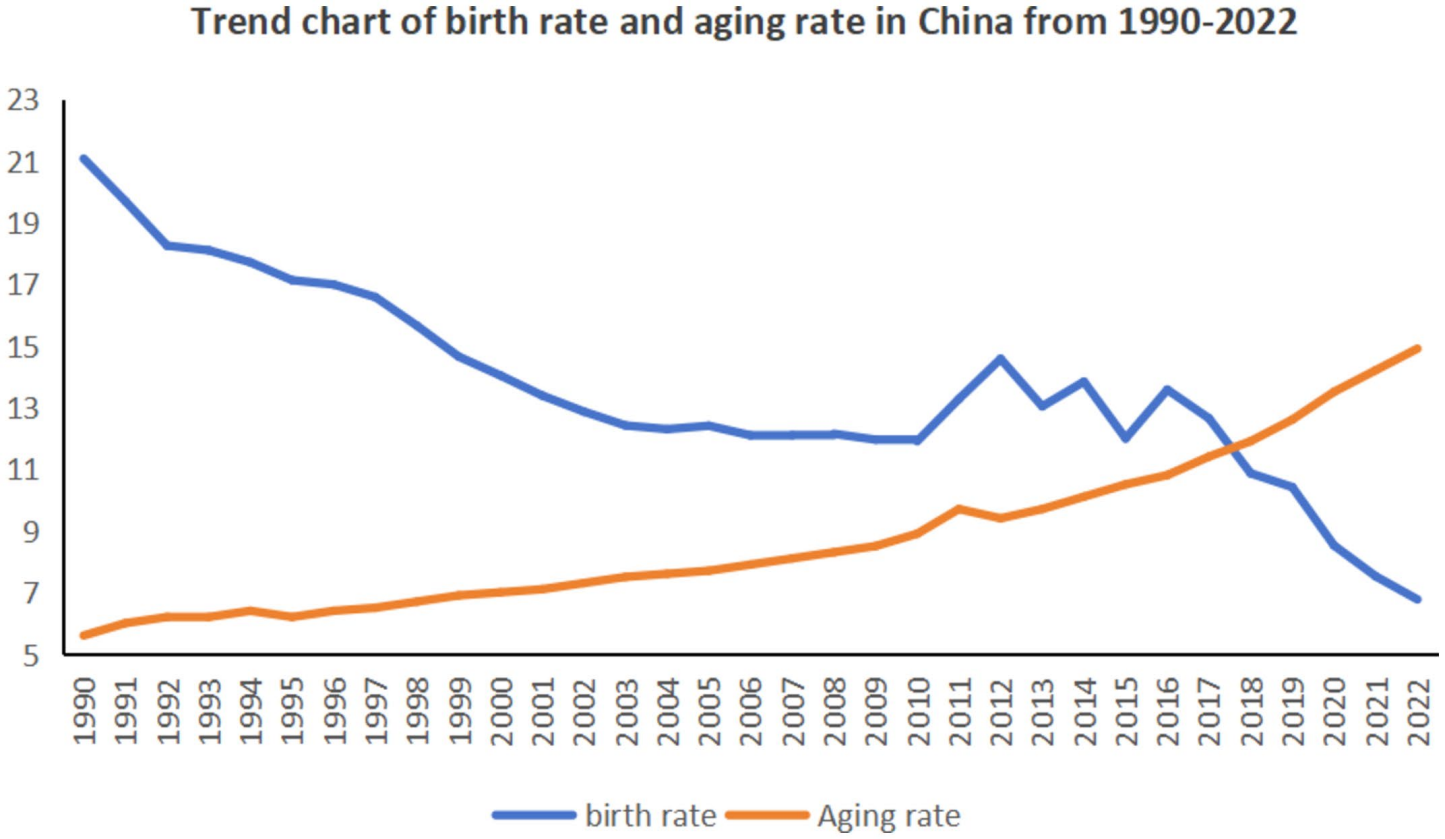
Trend of birth rate and aging rate in China from 1990 to 2022.

Since the implementation of the reform and opening-up policy in 1978, China’s economy has been effectively improved and developed (1), and has become the world’s largest developing country and the second largest economy in the world. However, the declining birth rate and the aging of China’s population mean that the demographic dividend that contributed to China’s rapid economic development is fast disappearing, the disappearance of the demographic dividend means an increase in labor costs, and companies may face difficulties in recruiting workers and rising labor costs, which will to some extent slow down the speed of economic growth. At the same time, population aging has intensified the burden of social older adult care, increasing expenditures on older adult care, medical care, and other aspects, putting pressure on the fiscal and social security systems. In addition, the decrease in the number of newborns has had an impact on areas such as education and consumption, and human capital has further affected the sustained growth of the economy (2). According to the China Statistical Yearbook, draw a trend chart of China’s birth rate and aging rate from 1990 to 2022, as shown in Figure 1.

As health is one of the important cornerstones of human capital, improving the health of the Chinese population is expected to increase China’s human capital (3). However, with the improvement of China’s economic level and the change of residents’ living habits, the prevalence of chronic diseases among Chinese residents is increasing year by year (4) and showing a younger trend. According to relevant data, the number of people suffering from chronic diseases in China is about 300 million, and the number of deaths due to chronic diseases accounts for 88% of the total number of deaths (5). This result has not only caused a serious burden on China’s health finance, but also brought serious harm to the quality and structure of China’s human capital. For this reason, the Chinese government has successively issued the “Healthy China 2030 Program” (6), “China’s Medium- and Long-Term Plan for the Prevention and Treatment of Chronic Diseases (2017–2025)” (7), aiming to improve the health level of residents from multiple perspectives, including the government, society and residents, etc. The improvement of the health level of the residents helps to improve the quality of human capital, and the optimization of the quality and structure of the human capital in turn promotes the economic development (8).

China put forward the “broadband China” strategy in 2013, and the scale of Chinese Internet users and Internet penetration rate have been rapidly improved. By 2021, China’s Internet development index has been second only to the United States, ranking second in the world. With the comprehensive popularization of Internet infrastructure and the improvement of the size of Internet users, all sectors of society began to integrate with the Internet in depth. In the field of medical treatment and health, with the combination of the Internet with medical treatment and health care, the use of online consultation, online payment and other means, on the one hand, not only reduces the time for residents to queue up, make appointments for doctors’ free time, offline payment and other steps, but also significantly reduces the time cost of residents. On the other hand, the use of the Internet enables residents to obtain more professional knowledge in medical treatment and health. According to the 49th Statistical Report on the Development of Internet in China, the number of Internet users using online medical care will increase from 21.7% in 2020 to 28.9% in 2021, with a growth rate of 38.7%. With the deep integration of the Internet with the health field and the medical field, residents can more easily access medical resources through the Internet, and can use the Internet to access medical knowledge without leaving home. The development of the Internet has made it more convenient for residents to access medical resources, and the cost of medical care has also dropped significantly. Can the Internet promote the health level of residents? In existing studies, it has been found that the health of the population is influenced by a number of factors, such as individual population factors, including age (9), gender (10), dietary habits (11), etc.; social factors such as environmental pollution (12), the use of renewable energy (13), the level of medical care in the region (14) and national policies (15) may all have an impact on the health of the population. Therefore, this study is based on the dual perspectives of sociology and economics, and based on the data from the 2021 Chinese General Social Survey (CGSS), investigate the effects of the frequency of Internet use and the economic income of Chinese residents on their health level from two dimensions. The data in this study are all from the 2021 Chinese General Social Survey (CGSS), and the Oprobit model is used to empirically test the effects of residents’ Internet usage frequency and economic income on residents’ health level, with the aim of providing data and policy recommendations for the Chinese government to build a “Healthy China” and improve the health level of the residents in the context of the new era. Compared with the existing literature, the possible marginal contributions of this study can be categorized into two parts: theory and reality:

First of all, in the existing literature, scholars have explored the factors that affect the health level of residents from multiple perspectives, but for the time being, there is no research that simultaneously brings the frequency of Internet use, economic income, and subjective health level of residents into the analysis framework. Therefore, based on the perspective of the Internet, this study also includes the frequency of Internet use, economic income and subjective health level of residents into the analytical framework, which is conducive to filling the existing research gaps and promoting the development of the Internet.

Secondly, this study uses the Oprobit model to conduct empirical research, and examines the impact of Internet use frequency on residents’ health level from the perspective of residents’ Internet use frequency and economic level. On the one hand, it supplements the existing research, and on the other hand, it provides data support for the Chinese government to promote the construction of a “healthy China” and the release of relevant policies, which is of practical significance.

Third, in the macro context of the gradual disappearance of the demographic dividend, which affects China’s economic development, investigating the impact of Internet use frequency on the health level of residents can not only explore the factors that affect the level of residents from the perspective of informatization, but also assess the significance of China’s Internet development.

Fourth, due to the urban–rural differences in China’s Internet development, in order to explore whether there are differences in residents’ registered residence registration in terms of Internet use frequency and economic income, this study divides residents according to rural registered residence registration and urban registered residence in the empirical test stage, and makes regression suggestions. The empirical test results help the Chinese government to improve the health level of China’s rural residents and urban residents in a targeted manner, and promote the balanced development of China’s economy.

Finally, this study will improve the credibility and validity of this study through the test of regional heterogeneity, the test of gender heterogeneity and the test of age heterogeneity, so as to provide data to help synchronize the improvement of the health level of the residents of different regions and different genders.

The remainder of the paper is structured as follows: section II is the literature review and hypothesis formulation, which organizes the existing literature and formulates the research hypotheses based on the existing literature. Section III is the research design, which explains the models, variables and data introduced in this study. Section IV is the empirical test and analysis of empirical results of this study. Section V and Section VI are related to the conclusion and discussion of this study, respectively.

## 2 Literature review and research hypothesis

### 2.1 Frequency of internet use and the health of the population

With China’s economic development and information technology development, China’s Internet business has also made great progress. According to the 49th Statistical Report on Internet Development in China, the number of Internet users in China reached 1.032 billion in December 2021, an increase of 42.96 million compared with December 2020, and the Internet penetration rate reached 73.0% (as this study adopts the data of the 2021 Chinese General Social Survey (CGSS), the total number of Internet users in China in 2021 is used in this part). In addition to this, the Report shows that the frequency of Internet use among Chinese netizens has shown an increase in *per capita* Internet time and a new trend of Internet use dominated by cell phones. Against this background, it is of great significance to explore the impact of Internet use frequency on residents’ self-assessed health level. Does Internet use affect the health of the population? At present, do not have a unified conclusion. Some studies believe that increasing the frequency of Internet use can promote the health of residents. From the perspective of social interaction, more and more scholars and doctors use the Internet to spread professional knowledge, and residents use the Internet to obtain professional health knowledge, thus affecting the health management of residents (16, 17). From the perspective of residents’ age, with the increase of residents’ age, social interaction may decline, but residents can improve the mental health of the older adult (18), improve their cognitive level (19), and reduce their sense of loneliness (20) through the use of various social software and video software in the Internet, thereby improving the mental health of residents. For example, the Rennoch study found that Internet use for social purposes may help older adults reduce loneliness and depressive symptoms (21). Yu Xinfang used data from the 2016 and 2018 China Social Tracking Survey of the Older Adult and found that Internet use improves cognitive functioning in older adults using ordinary least squares and mediation analysis, but there are urban–rural differences (22). Guo, based on a total of four periods of Chinese household tracking survey data in 2014, 2016, 2018, and 2020, empirically examined that Internet use can promote physical health of older adults, and there is heterogeneity in gender, region, etc. (23) But does the increase in the frequency of Internet use always promote the health of residents? Some scholars have found that Internet use will also have a negative impact on residents’ health. From the perspective of residents’ health, due to the radiation brought by the Internet and the need for residents to maintain a posture for a long time in the process of using the Internet, residents may have headaches, backache, finger numbness and other problems (24). From the perspective of mental health, excessive or even addicted use of the Internet may lead to residents’ Internet addiction, which in turn will affect residents’ social interaction in real life, thus improving residents’ sense of loneliness, thus affecting residents’ mental health (25). Hamilton, in order to explore whether Internet use protects the mental health of older adults, empirically tested the results using ordinary least squares regression model based on data from the 2016 Chinese General Social Survey, which showed that older adults who use the Internet regularly are more likely to be depressed than those who do not use the Internet regularly (26). Scholars have explored the impact of residents’ frequency of Internet use on residents’ health level from different angles of entry, and although there are differences in the results of the study, they all confirm that the frequency of residents’ Internet use affects residents’ health. Based on this, this study proposes the first research hypothesis:

*H1:* Internet use has a significant effect on residents’ self-rated health.

### 2.2 Economic income and health of the population

In existing research on residents’ economic income and health level, most of the results indicate that residents’ economic income can positively affect their health level. From the perspective of economic income, residents with higher incomes are able to obtain nutrients that better meet their health needs (27), and their quality of life also varies with differences in economic level (28). Moreover, as the income gap widens, the income gap among residents will further lead to an imbalance in their health levels (29). Also based on the income perspective, higher-income residents are able to improve their living environment through the economy (30), improved their diets (31), improved treatment of diseases (32), reduced prevalence of disease (33) and reduced loneliness (34). In addition, from the perspective of education, economic income can affect residents’ education level, and different levels of education experience also have differences in residents’ health level (35). The higher level of education has a greater impact on health beliefs (36). Based on this, the second research hypothesis of this study is proposed:

*H2:* Increased economic income contributes to the health of the population.

### 2.3 There are urban–rural differences in the impact of residents’ frequency of internet use and economic income on their health

With China’s rapid economic development, the gap between urban and rural areas in China has further narrowed, but there are still some differences between urban and rural areas in terms of infrastructure, economic development, and education level, so there may be urban–rural differences in the frequency of Internet use and the impact of economic income on the health of the residents. Based on data from the 2017 Chinese General Social Survey (CGSS), Xu Jinyan explored the relationship between Internet use and residents’ mental health, and found that there are urban–rural differences (37). Han Tanqian has similar findings (38). Liu Weizhong, on the other hand, found that differences in socioeconomic status have different impacts on the physical and mental health of urban and rural residents (39). Based on this, the third hypothesis analysis is proposed in this study:

*H3:* There are urban–rural differences in the impact of frequency of Internet use and economic income on the health of the population.

## 3 Study design

### 3.1 Description of variables

#### 3.1.1 Explained variables

The explained variable of this study is the self-assessed health level (Health) of the population, based on the collection of data on what residents say about their health in the Chinese General Social Survey (CGSS) program team. The question on residents’ health level in the 2021 Chinese General Social Survey (CGSS) is described as follows:. What do you think is your current state of health? There are five options: very unhealthy, relatively unhealthy, average, relatively healthy, and very healthy; based on the order of the options, the above five options are assigned a value of 1–5, with 1 representing very unhealthy and 5 representing very healthy, with the larger the score, the higher the self-assessed health level of the residents.

#### 3.1.2 Explanatory variables

The explanatory variables of this study are the frequency of residents’ Internet use and residents’ economic income level. First of all, as for the selection of the indicator of residents’ Internet usage frequency (Internet), in the empirical test, different scholars have different selections of this indicator, as this study is based on the 2021 Chinese General Social Survey (CGSS), the question about residents’ Internet usage frequency in this survey is described as follows: In the past year, how much did you use the Internet (including accessing the Internet with your cell phone)? There are five corresponding options, namely, never, seldom, sometimes, often, and very often. Based on the order of the options, the above five options are assigned a value of 1–5, with 1 representing never using the Internet and 5 representing very frequent use of the Internet, and the larger the score, the higher the frequency of Internet use. Then it is the determination of residents’ economic income (Income) indicators, still using CGSS data, in the survey on residents’ economic income questions described as follows: What was your personal total income for the whole of last year? In the empirical evidence, the residents’ economic income is measured using logarithmization.

#### 3.1.3 Control variables

Based on the finding that the factors affecting the health level of residents are becoming more and more diversified, this study introduces control variables reflecting residents’ personal characteristics, social characteristics and family characteristics. The control variables in this study are as follows:

1. Sex (Sex), the reason for sex may lead to differences in the physical fitness of the residents, so this study included sex as a control variable, where males were assigned a value of 1 and females were assigned a value of 0.
2. Age (Age), the variable of age was controlled for as the health level of the population varies by age.
3. Education level (Educate), in the existing research found that the level of education of the residents will affect the health level of the residents, so this study controls the variable of education level of the residents. According to the question in the questionnaire, “What is your current highest level of education?” The results of the answer were assigned. The value of never having been to school is 0, illiterate or semi-illiterate is 1, elementary school is 6, junior high school is 9, high school/secondary school/technical school/vocational high school is 12, junior college is 15, undergraduate degree is 16, and master’s or doctoral degree is 19.
4. Marital status (Ms), whether or not one is already married may also have an effect on the health level of the residents, so this study introduces the marital status of the residents and controls for it, assigning a value of 1 if the residents are married or cohabiting; and 0 for others, with others representing unmarried, divorced, widowed, and separated.
5. Hukou (Hr), because of the disparities in infrastructure, education, and medical care between rural and urban China, the health level of rural and urban residents may be different. It is assigned a value of 1 if it is an agricultural household and 0 if it is a non-agricultural household.
6. Physical activity (exercise), with different frequencies, also affects the health level of the population, so it will be controlled for in the empirical study.
7. Socialization of the population, the level of socialization at different frequencies may also have an impact on the health of the residents, which is divided into two variables in this variable, Socializing with Neighbors (SWN) and Socializing with Friends (SWF).
8. Purchase of medical insurance, China is a country with a relatively well-developed medical insurance system, and medical insurance is, to a certain extent, a kind of social support for the health level of the population. In this variable is divided into two variables, which are social medical insurance (SMI) and commercial medical insurance (CMI).
9. Family Economic Income (F-Income): Generally speaking, high-income families have more diverse choices in terms of lifestyle, quality of life, work and living environment, medical security, education level, etc. than low-income families, which affects the health level of family members. Therefore, this article introduces the variable of family economic income and logarithmizes it in empirical research.

### 3.2 Data sources

The CGSS database is the earliest national, comprehensive, and continuous academic survey project conducted in China. This study used the 2021 Chinese General Social Survey (CGSS) data and based on the self-assessment of residents’ health levels in this dataset, asked: What do you think is your current physical health status?, Respondents can answer based on their actual level of physical health, with a total of five corresponding options: 1-very unhealthy, 2-relatively unhealthy, 3-average, 4-relatively healthy, and 5-very healthy. Of course, respondents can also choose “do not know” or “refuse to answer.” After excluding missing variables and respondents who refused to answer, this study obtained 4,487 observations, indicating a certain representativeness of the research sample. Part of the data in this study was logarithmically processed during the sorting process.

Specific definitions of variables and descriptive statistics are shown in Table 1. According to the descriptive results in Table 1, the mean value of Chinese residents’ self-assessed health level is 3.524, which is close to the level of “relatively healthy,” but there is still a certain gap from the level of “very healthy.” At the same time, the average value of residents’ Internet use frequency is 3.455, slightly higher than “sometimes” among the five levels of Internet use, but still far from the level of “frequent Internet use.” In addition, in the descriptive statistics of the variable of residents’ economic income, the minimum value of residents’ economic income is 5.644, and the maximum value is 23.252, which also shows that there is a big gap between the economic levels of Chinese residents.

**Table 1:** Descriptive statistics of variables.

Variant	Sample size	Average value	(Statistics) standard deviation	Minimum value	Maximum values
Level of health	4,487	3.524	1.053	1	5
Frequency of Internet use (Internet)	4,487	3.455	1.621	1	5
Economic income (Income)	4,487	14.684	1.924	5.644	23.252
Gender (Sex)	4,487	0.51	0.5	0	1
Age	4,487	52.165	16.148	18	99
Level of education (Education)	4,487	9.696	4.567	0	19
Marital status (MS)	4,487	0.78	0.414	0	1
Domicile (Hr)	4,487	0.542	0.498	0	1
Physical exercise (exercise)	4,487	2.929	1.606	1	5
Socializing with neighbors (swn)	4,487	3.702	2.191	1	7
Socializing with friends (swf)	4,487	3.836	1.884	1	7
Social Medical Insurance (SMI)	4,487	0.955	0.207	0	1
Commercial Medical Insurance (CMI)	4,487	0.161	0.368	0	1
Family Economic Income (F-Income)	4,487	15.745	1.805	3	23.249

### 3.3 Modeling

In the model setting for the empirical test, reference is made to Fei (40) and Mang’s (41) study, a benchmark model (Equation 1) for identifying the impact of factors such as residents’ Internet usage frequency and economic income on residents’ health level is constructed as follows:


(1) $${\mathrm{Health}}_{i}=\alpha {\mathrm{Internet}}_{i}+\beta {\mathrm{Income}}_{i}+\delta {\mathrm{CV}}_{i}+\varepsilon_{i}$$

Where ${\mathrm{Health}}_{i}$ represents the self-rated health level of the ith sample, the ${\mathrm{Internet}}_{i}$ represents the frequency of Internet use of the ith sample, the ${\mathrm{Income}}_{i}$ represents the economic income of the ith sample. Also ${\mathrm{CV}}_{i}$ represents the control variables of the ith sample; α,β, and δ then represent the regression coefficients of the variables of interest; ε is the attendant disturbance term of the model.

Since individual-level variables such as residents’ self-assessed health level and frequency of Internet use are ordered response variables, Oprobit model will be used for testing in the empirical study. Meanwhile, in order to avoid the influence of outliers on the empirical test results, all continuous number variables are Winsorized before the empirical test.

## 4 Empirical results and analysis

### 4.1 Analysis of the results of the empirical tests of the benchmark regression

In order to specifically examine the mechanism of the role of residents’ frequency of Internet use and economic income on health level, this study is based on the basic model (1), using the Oprobit model to divide the sample into the full sample, towns and villages, and introduce the two variables of residents’ frequency of Internet use and economic income in batches for regression tests, and the detailed regression results are shown in Table 2.

**Table 2:** Benchmark regression results.

Variant	Health								
	Full sample	Cities and towns	Countryside	Full sample	Cities and towns	Countryside	Full sample	Cities and towns	Countryside
	Model 1	Model 2	Model 3	Model 4	Model 5	Model 6	Model 7	Model 8	Model 9
Internet	0.064*** (4.80)	0.057*** (2.88)	0.065*** (3.61)				0.059*** (4.44)	0.054*** (2.69)	0.060*** (3.34)
Income				0.059*** (4.55)	0.027 (1.16)	0.066*** (4.11)	0.054*** (4.16)	0.024 (1.06)	0.062*** (3.86)
Sex	0.09*** (2.78)	0.074 (1.54)	0.109** (2.43)	0.057* (1.73)	0.057 (1.19)	0.065 (1.41)	0.065* (1.97)	0.069 (1.39)	0.07 (1.53)
Age	−0.015*** (−10.16)	−0.016*** (−7.73)	−0.015 (−7.10)	−0.017*** (−12.28)	−0.018*** (−9.33)	−0.016*** (−8.22)	−0.015*** (−9.77)	−0.015*** (−7.97)	−0.013*** (−6.10)
Education	0.012** (2.43)	0.007 (0.90)	0.019*** (2.79)	0.013*** (2.64)	0.009 (1.19)	0.02*** (3.06)	0.01* (1.91)	0.003 (0.37)	0.017** (2.51)
MS	0.01 (0.25)	−0.042 (−0.74)	0.075 (1.32)	0.016 (0.40)	−0.031 (−0.55)	0.072 (1.27)	0.005 (0.12)	−0.037 (−0.65)	0.059 (1.04)
Hr	0.113*** (2.89)			0.13*** (3.29)			0.14*** (3.54)		
Exercise	0.043*** (4.00)	0.075*** (4.61)	0.018 (1.24)	0.045*** (4.26)	0.079*** (4.85)	0.019 (1.38)	0.042*** (3.93)	0.074*** (4.57)	0.017 (1.17)
swn	0.025*** (2.94)	0.023* (1.86)	0.028** (2.35)	0.027*** (3.21)	0.024* (1.91)	0.03** (2.56)	0.027*** (3.20)	0.025** (2.01)	0.03** (2.53)
swf	0.039*** (3.85)	0.06*** (3.84)	0.025* (1.88)	0.041*** (3.99)	0.062*** (3.97)	0.026* (1.96)	0.037*** (3.64)	0.057*** (3.69)	0.023* (1.67)
SMI	−0.126 (−1.62)	−0.052 (−0.43)	−0.178* (−1.76)	−0.118 (−1.53)	−0.047 (−0.38)	−0.162 (−1.61)	−0.124 (−1.60)	−0.055 (−0.45)	−0.165 (−1.63)
CMI	0.045 (0.97)	0.068 (1.12)	0.039 (0.54)	0.038 (0.83)	0.068 (1.12)	0.032 (0.44)	0.032 (0.70)	0.054 (0.89)	0.025 (0.35)
F-Income	0.079*** (7.06)	0.058*** (2.85)	0.082*** (5.89)	0.052*** (3.93)	0.046* (1.90)	0.054*** (3.37)	0.049*** (3.71)	0.040* (1.67)	0.051*** (3.17)
N	4,487	2055	2,432	4,487	2055	2,432	4,487	2055	2,432
R-squared	0.062	0.057	0.068	0.062	0.055	0.069	0.064	0.056	0.070

t-values in parentheses, ***, **, and * indicate significant at the 1, 5, and 10% levels, respectively.

Table 2 reflects the mechanism of urban and rural residents’ Internet use frequency and economic income on self-assessed health level. Among them, model 1, model 2 and model 3 test the effect of residents’ Internet usage frequency on residents’ health level in the whole sample, urban and rural areas respectively; model 4, model 5 and model 6 test the effect of residents’ economic income on health level; and model 7, model 8 and model 9 test the effect of residents’ Internet usage frequency and economic income on health level at the same time.

First, the effect of residents’ Internet use frequency on health level is analyzed. According to the regression test results of model 1, model 2 and model 3, it can be seen that the frequency of Internet use passes the 1% significance level test in the regression of the full sample, urban and rural samples, with the coefficients of 0.064, 0.057, and 0.065, respectively, with the highest coefficients in the regression results in the rural sample. This result indicates that the frequency of residents’ Internet use can positively promote the health level of residents, which verifies the correctness of hypothesis 1. At the same time, the regression coefficient of rural residents’ Internet usage frequency is higher than that of urban residents, indicating that rural residents’ Internet usage frequency promotes health level higher than that of urban residents, which preliminarily verifies that there are urban–rural differences in the influence of residents’ Internet usage frequency on health level.

Secondly, the impact of residents’ economic income on the level of health level is analyzed. According to the regression test results of model 4, model 5 and model 6, it can be seen that residents’ economic income passes the 1% significance level test in both the full-sample model and the rural sample model, and the coefficients are 0.059 and 0.066 respectively, which indicates that residents’ economic income is positively correlated with the level of residents’ self-assessed health. According to the regression results of the full sample can initially verify the correctness of hypothesis 2, while in the regression model of the urban sample, the results show that there is no significant relationship between residents’ economic income and self-assessed health. This conclusion can initially verify the correctness of hypothesis H3 that there are urban–rural differences in the impact of residents’ economic income on health.

Third, after considering the effects of residents’ Internet usage frequency and economic income on residents’ self-assessed health level separately, Model 7, Model 8 and Model 9 consider the effects of Internet usage frequency and economic income on residents’ health level comprehensively. Comprehensive comparison of the regression results of Model 1-Model 9 shows that although the regression coefficients have all slightly decreased, the regression coefficient of residents’ Internet use frequency is still positive; the regression coefficients of economic income are also significantly positive in both the full sample and the rural sample, but the regression results of the urban sample are the same as those of Model 5, which shows that there is no significant relationship between the residents’ economic income and the self-assessed health. According to the empirical results of model 7, model 8 and model 9, the correctness of hypothesis 1, hypothesis 2 and hypothesis 3 can be verified together.

Finally, the regression results of the control variables are synthesized and analyzed. In the full sample, residents’ gender, education level, frequency of physical activity, socializing with neighbors, socializing with friends, and household economic income all have a positive and significant effect on residents’ health level. There are several possible reasons for this, firstly, as the level of education of residents increases, residents are more able to respond to physical and mental illnesses in a timely manner, thus nipping illnesses in the bud and contributing to a positive growth in health levels, a finding that is consistent with the Veladas (42); secondly, as the frequency of residents’ physical activity increases, it can improve residents’ physical fitness and thus improve their health level, this conclusion is consistent with Li Cong’s findings (43), while Li Cong’s study found that urban residents’ physical activity has a more health-promoting effect than that due to rural residents, comparing Model 2 and Model 3, it can be seen that the regression results of urban residents’ physical activity on their health level are significant, while the results of rural samples are not significant, the conclusion is similar to Li Cong’s findings. Third, social interaction can promote residents’ health level, probably because residents’ social interaction with neighbors and friends reduces their loneliness and anxiety, which improves their mental health level, and the mental health level can positively affect residents’ physical health level. Xinyue (44) also has a similar research finding. Finally, in the regression results, the residents’ physical age is negatively correlated with their health level, which is well understood, as age increases, the residents’ physical fitness and mental health level decreases accordingly, thus showing a negative correlation in the baseline regression. In the urban sample, physical exercise, residents’ social interactions, and household economic income are able to promote the growth of residents’ health levels, possibly for reasons similar to those in the full sample. In the rural sample, residents’ health level is positively correlated with education level, social intercourse, and household economic income, and it is worth noting that the frequency of residents’ physical exercise and residents’ health level are not significant in the rural sample, the possible reason is that compared to urban residents, rural residents are more engaged in physical labor. Rural residents earn income through a large amount of physical labor, and their physical fitness is higher than that of urban residents in daily physical work. Rural residents’ physical work replaces urban residents’ physical exercise, so the frequency of physical exercise for rural residents is not significant in the regression analysis.

The empirical findings of this study show that the frequency of residents’ Internet use has a significant positive contribution to residents’ health. The possible reasons for this are that, on the one hand, with the improvement of digital technology, residents can obtain professional and scientific health knowledge through the Internet, such as exercise knowledge provided by professional coaches, healthy diet programs provided by nutritionists, and health care knowledge provided by medical personnel, etc., so that residents all over the country can improve their health by obtaining professional knowledge on exercising, diet, and health care that suits their own needs; At the same time, through the construction of telemedicine, Internet medical consultation and services by the government, hospitals and other relevant departments, residents can obtain the help of medical professionals through the Internet, helping residents to further understand their own health level, thus promoting the healthy development of residents’ health level. Moreover, since China is a country with large differences in regional economic development, the level of medical care also varies from region to region and from province to province. Through the Internet’s telemedicine, online consultation and online medical consultation and services, it can help residents in backward areas to obtain higher quality medical services and medical assistance, thus helping to promote the simultaneous growth of the health level of residents in different regions. On the other hand, as China’s urbanization process accelerates, residents of all strata, both men and women, are subject to greater pressure of life and work. Therefore, by using the Internet, adopting a low-cost way of interpersonal communication, reading news and novels of interest to residents, watching movies and short videos, and shopping online, residents can not only release their own pressure and thus reduce the sense of loneliness, but also obtain positive emotional incentives through the Internet, thus helping to promote the synchronous growth of the health level of residents in different regions. This finding is consistent with Rennoch (21), Guo (23). But in this study, we found that the frequency of Internet use has a more significant effect on rural residents, the possible reason is that compared with rural residents, urban residents have better medical services, infrastructure and professional help, so rural residents can better improve their own health through the Internet.

In addition to this, the empirical findings show that the economic income of the population can promote the health level of the population, this is similar to the findings of Sanders (31), Michael (32) and other scholars, which may be due to the fact that as the economic level of the residents increases, the residents are able to improve their own health level by improving the living environment, improving the diet level and reducing the disease rate, and so on. Meanwhile, in the analysis of urban–rural differences, it was found that the effect of economic level on the health level of urban residents was not significant. This may be due to the fact that urban areas have better infrastructures, more hospitals, higher ratios of medical personnel, and better quality of medical services, and urban residents can obtain sufficient medical services with lower economic expenditures, so economic income does not affect the health of urban residents. In rural areas, however, compared to urban areas, a higher level of economic expenditure is needed to obtain adequate medical services, nutritional advice, and so on. Therefore, increasing the economic income of rural residents positively affects the level of health of the population.

### 4.2 Robustness tests

In order to further improve the credibility and robustness of the empirical test, this study adopts the following two methods that will be used for the robustness test, which are the way of excluding some samples from special areas, and the way by replacing the model.

#### 4.2.1 Robustness test for excluding exceptional samples

Among the data obtained from 19 provincial cities, two municipalities, Beijing and Chongqing, are included. Due to the special status of municipalities, both the economic development, infrastructure construction, and medical level will be higher than that of other provinces, therefore, in this part, in order to avoid the interference of the special status of municipalities on the regression results, Beijing and Chongqing are excluded for the robustness test. The results of the robustness test excluding the special samples are shown in Table 3. According to the regression results in Table 3, the regression coefficients of the full sample and the rural sample have slightly increased compared with Table 2, and the regression coefficients of the residents’ Internet usage rate and economic income have passed the significance level test of 1%, and the coefficients are positive, which indicates that the residents’ Internet usage rate and economic income still have a significant role in the promotion of the residents’ health level. In the urban sample, probably due to the exclusion of special samples, the frequency of residents’ Internet use only passes the 5% significance level test, but the coefficient is also positive, which also proves that even in the urban sample, the rate of residents’ Internet use has a positive impact on residents’ health level. This finding proves that the results of the underlying regression are very robust.

**Table 3:** Robustness test results.

Variant	Robustness test for excluding exceptional samples			Robustness tests using Ologit models		
	Full sample	Cities and towns	Countryside	Full sample	Cities and towns	Countryside
Internet	0.063*** (4.18)	0.055** (2.37)	0.067*** (3.71)	0.107*** (4.59)	0.097*** (2.78)	0.111*** (3.52)
Income	0.058*** (3.94)	0.028 (1.00)	0.066*** (3.35)	0.092*** (3.97)	0.033 (0.82)	0.106*** (3.69)
Control variable	Containment	Containment	Containment	Containment	Containment	Containment
*N*	3,516	1,480	2036	4,487	2055	2,432
R-squared	0.064	0.061	0.067	0.064	0.056	0.070

t-values in parentheses, ***, **, and * indicate significant at the 1, 5, and 10% levels, respectively.

#### 4.2.2 Robustness tests for replacement models

In this part, Ologit model is used to replace Oprobit for the test, and the regression results are shown in Table 3. Comparing the regression results based on Ologit model in Table 3 with those based on Oprobit model in Table 2, it can be seen that the regression coefficients values based on the Oogit model are all increased to different degrees, indicating that the increase of residents’ Internet use rate and economic income both can significantly promote the residents’ health level, and the regression results are very robust.

### 4.3 Heterogeneity test

Based on the above empirical test, although residents’ Internet use frequency, economic income and residents’ health level have a significant positive impact, it does not explore the heterogeneity of the impact of Internet use frequency on different groups of residents. There is a regional imbalance in China’s economic development. As far as network infrastructure is concerned, there is a big difference between the eastern, western and central regions, and the Internet penetration rate between urban and rural areas is also unequal. Moreover, there are significant differences in Internet use among different gender groups and different age groups. Therefore, it is of great practical significance to explore the heterogeneity of Internet use frequency on residents’ health level. In this section, we will empirically test whether region, gender, and age affect residents’ health levels.

#### 4.3.1 Tests for regional heterogeneity

Table 4 shows the results of the heterogeneity test by region. According to the regression results in Table 4, it can be seen that there is a positive and significant effect of the frequency of Internet use on residents’ self-assessed health in the full sample, in which the regression coefficients are consistent between the east and the west, and the lowest in the center. However, in the regression results by urban and rural areas, there are differences. In the eastern region, the frequency of Internet use by urban residents promotes the health level of residents more than that in rural areas, which is different from the results of the benchmark regression. In the central and western regions, however, the results of the effect of the frequency of Internet use on the health level of urban residents are not significant, while it is significant for the health level of rural residents. This finding also verifies the correctness of hypothesis 3. For the effect of residents’ economic income and residents’ health level, there are also regional and urban–rural differences. In the full sample, the highest level of impact of residents’ economic income on residents’ health level is found in the eastern region and the second in the central region, while the impact of residents’ economic income on health level in the western region does not pass the significance level test. From the urban and rural samples, the economic income of residents can promote the health level of residents in the rural samples in the eastern region and the western region, but it is not significant in the urban samples. In the western region, the effect of residents’ economic income on the promotion of health level is not significant in both urban and rural samples. This finding once again verifies the correctness of Hypothesis 3 that there are urban–rural differences in the health promotion effect of residents’ economic income.

**Table 4:** Tests for regional heterogeneity.

Variant	Health								
	Eastern part			Central Region			Western region		
	Full sample	Cities and towns	Countryside	Full sample	Cities and towns	Countryside	Full sample	Cities and towns	Countryside
Internet	0.067*** (3.48)	0.064** (2.58)	0.059* (1.91)	0.052** (2.16)	0.049 (1.13)	0.050* (1.70)	0.067** (2.29)	0.028 (0.52)	0.082** (2.34)
Income	0.062*** (3.06)	0.044 (1.41)	0.064** (2.32)	0.046** (2.01)	−0.033 (−0.69)	0.067** (2.48)	0.036 (1.37)	0.077 (1.46)	0.028 (0.89)
Control variable	Containment	Containment	Containment	Containment	Containment	Containment	Containment	Containment	Containment
*N*	2,245	1,327	918	1,323	434	889	919	294	625
R-squared	0.060	0.052	0.075	0.063	0.068	0.063	0.081	0.080	0.079

t-values in parentheses, ***, **, and * indicate significant at the 1, 5, and 10% levels, respectively.

#### 4.3.2 Tests for gender heterogeneity

Table 5 shows the results of the heterogeneity test by gender. According to the regression results in Table 5, it can be seen that in the full sample, the frequency of Internet use and the economic income of residents have a promoting effect on the health level of male residents and female residents, but the frequency of Internet use promotes the health level of female residents more than that of males, while the economic income promotes the health level of male residents more than that of females. In the urban–rural difference, for males, the frequency of Internet use and economic income are both significant for the health level of rural residents, and the regression of the two variables on the health level of urban residents fails the significance test. For females, there is a difference in that the frequency of Internet use contributes more to the level of health for urban residents than for rural residents.

**Table 5:** Gender heterogeneity test.

Variant	A male			Females		
	Full sample	Cities and towns	Countryside	Full sample	Cities and towns	Countryside
Internet	0.053*** (2.87)	0.041 (1.45)	0.061** (2.45)	0.072*** (3.73)	0.074*** (2.60)	0.062** (2.36)
Income	0.070*** (3.54)	0.021 (0.58)	0.087*** (3.58)	0.046*** (2.66)	0.030 (1.00)	0.049** (2.24)
Control variable	Containment	Containment	Containment	Containment	Containment	Containment
*N*	2,290	1,022	1,268	2,197	1,033	1,164
R-squared	0.056	0.043	0.066	0.075	0.075	0.080

t-values in parentheses, ***, **, and * indicate significant at the 1, 5, and 10% levels, respectively.

#### 4.3.3 Tests for age heterogeneity

The World Health Organization classifies less than 45 years of age as youth and more than 45 years of age as middle-aged and old-aged, and based on this, 45 years of age was set as the cut-off for the age heterogeneity test in this study. Table 6 shows the results of the heterogeneity test by gender. From the regression results in Table 6, it can be seen that the health impact of the frequency of Internet use on the full sample, urban residents and rural residents has a promoting effect in all age groups, in which the frequency of Internet use has a more significant promoting effect on the self-assessed health of residents aged 45 years old and below. As for the relationship between economic income and residents’ health level, residents’ economic income passed the significance test for the health level of residents over 45 years old. However, for residents under the age of 45, the coefficient is not significant and negative, indicating that based on the data from this study, the economic income of residents under the age of 45 has a relatively small inhibitory effect on their health. The possible reason for this inhibitory effect is that residents may work excessively, take part-time jobs, or even sacrifice their health in order to improve their economic level, in order to pay for the cost of living and various expenses of their families from the beginning of entering society, resulting in a negative coefficient in the test.

**Table 6:** Age heterogeneity test.

Variant	Residents aged 45 and under			Residents over 45 years of age		
	Full sample	Cities and towns	Countryside	Full sample	Cities and towns	Countryside
Internet	0.166*** (4.62)	0.154** (2.57)	0.168*** (3.68)	0.066*** (4.81)	0.071*** (3.50)	0.059*** (3.16)
Income	−0.021 (−0.88)	−0.042 (−1.19)	0.002 (0.06)	0.088*** (5.57)	0.071** (2.27)	0.091*** (4.96)
Control variable	Containment	Containment	Containment	Containment	Containment	Containment
*N*	1,568	737	831	2,919	1,318	1,601
R-squared	0.018	0.024	0.023	0.040	0.038	0.040

t-values in parentheses, ***, **, and * indicate significant at the 1, 5, and 10% levels, respectively.

## 5 Conclusion and discussion

### 5.1 Conclusion

Based on the data from the 2021 Chinese General Social Survey (CGSS), this study examines the effects of residents’ frequency of Internet use and economic income on residents’ self-assessed health level based on the dual perspectives of sociology and economics. The results of the empirical study show that: first, the frequency of residents’ Internet use has a significant positive promotion effect on residents’ health level, while the effect is more significant in the rural sample; second, the economic income of residents can significantly promote the health level of residents in both the full sample and the rural sample, but the effect of the economic income of urban residents on the health level of residents does not pass the significance test. Third, in the robustness test of excluding special regional samples and replacing the model, the results of the robustness test are largely consistent with the results of the benchmark regression. Fourth, in the heterogeneity test, it can be seen that there are regional differences, gender differences and age differences in the effects of residents’ Internet use frequency and economic income on residents’ health level. In the regional heterogeneity test, the frequency of residents’ Internet use has the most significant effect on residents’ health in the eastern and western regions, and last in the center; while the promotion effect of economic income on residents’ health level is most obvious in the eastern region, second in the center, and third in the west. In the test of gender heterogeneity, the frequency of Internet use has a more significant effect on the health of female residents, while economic income has a more significant effect on the health of male residents. In the age heterogeneity test, the frequency of Internet use has a more significant effect on the health level of residents under 45 years old, while economic income has a more significant effect on the health level of residents over 45 years old.

### 5.2 Discussion

This research adopts empirical research, and at the same time brings the frequency of Internet use, economic income and self rated health level of residents into the research framework. The empirical research results, on the one hand, supplement the existing literature, on the other hand, can provide data support for the Chinese government to promote the construction of a “healthy China” and the release of relevant policies from the data level, and also provide social data support for the significance of China’s Internet development, which is of practical significance. Although this study can supplement existing literature from a theoretical perspective and provide data support for China’s strategic development from a practical perspective, it still has certain limitations.

Firstly, limited by the availability of data, this study used residents’ self-rated health level data and did not further investigate residents’ physical and mental health separately. Secondly, the health level of residents is influenced by many factors, and this study did not further explore possible influencing factors, which is also an issue that needs to be improved in future research. Finally, although this study has separately explored the impact of residents’ Internet use frequency and economic income on residents’ health level, the mechanism of residents’ health impact needs to be further explored.

In further research, we hope to analyze whether the frequency of Internet use and residents’ economic income can have a positive impact on the above two aspects by exploring residents’ physical and mental health, respectively.

## 6 Recommendations

Based on the above conclusions, the following recommendations are made:

First, further increase China’s Internet penetration rate, especially in rural areas. Since the findings of this study indicate that the frequency of Internet use by residents can promote the health level of residents, which is one of the important cornerstones of human capital, advancing the health of the population can enhance China’s human capital, thus promoting the benign enhancement of China’s economy. However, China’s Internet penetration rate still needs to be improved. As of December 2023, China’s Internet penetration rate was 77.5%, which is still a certain gap with the Internet penetration rate of developed countries, so China still needs to further improve. Meanwhile, the Internet penetration rate in urban areas is 83.3%, and the Internet penetration rate in rural areas is 66.5%, and there is still a gap between urban and rural Internet penetration rates. Therefore, the Internet penetration rate in rural areas can be improved through measures such as improving basic network facilities and expanding Internet application scenes.

Secondly, training in specialized Internet skills. As of December 2023, the size of China’s non-Internet users is 317 million, of which the resident population in rural areas is the main reason why non-Internet users do not use the Internet, such as the lack of practical skills and the age factor. Therefore, government departments can improve the level of Internet use skills of non-Internet users by organizing professionals to go to the countryside to carry out public welfare lectures and Internet skills training courses, so as to expand the population of Internet users, which on the one hand, can further increase the Internet penetration rate in China; At the same time, after mastering Internet skills, this group of people can also access specialized knowledge and high-quality medical services through the Internet, thus improving their own health, and thus alleviating the health inequality caused by the income gap between urban and rural residents and the difference in infrastructure.

Finally, promote the high-quality development of Internet content. As Internet penetration increases, the content disseminated on the Internet is a mixture of good and bad, and in order to prevent negative and unhealthy information from misleading Internet users, the relevant government departments should strengthen the positive guidance and supervision of Internet content to avoid the adverse effects of negative content. Further, government departments can organize universities, hospitals and other relevant institutions to use Internet tools to disseminate content such as high-quality videos and scientific health knowledge. At the same time, the government also needs to strengthen the ability of residents to screen information on the Internet, so as to avoid the physical and mental health of residents being affected by wrongly trusting false and unhealthy information.

## Data Availability

The original contributions presented in the study are included in the article/Supplementary material, further inquiries can be directed to the corresponding author.
